# A Narrative Review of Early Pregnancy Diagnosis Technologies for Livestock: From Conventional to Intelligent Systems

**DOI:** 10.3390/ani16182897

**Published:** 2026-09-15

**Authors:** Yang Shen, Yujie Zhang, Junyi Meng, Yutong Han, Jitong Xu, Hongying Wang, Liangju Wang

**Affiliations:** College of Engineering, China Agricultural University, Beijing 100083, China; shenyang0515@cau.edu.cn (Y.S.); 18300727091@163.com (Y.Z.); 2023307140112@cau.edu.cn (J.M.); hanyutong0555@cau.edu.cn (Y.H.); xujitong0718@cau.edu.cn (J.X.); hongyingw@cau.edu.cn (H.W.)

**Keywords:** early pregnancy diagnosis, livestock, biomarkers, imaging techniques, spectroscopy, artificial intelligence

## Abstract

Accurate and efficient early pregnancy diagnosis (EPD) in livestock is crucial for optimizing breeding management and enhancing productivity in modern animal husbandry. Over the past century, EPD technology has evolved from empirical methods to sophisticated techniques, encompassing biochemical marker detection, ultrasonic imaging, and further extending to emerging non-invasive approaches such as infrared thermography (IRT) and spectroscopic analysis. These advancements have not only improved diagnostic accuracy but also broadened the research scope to include small livestock and multiple species. This review critically examines the historical evolution, current methodologies, and applications of EPD technology, with a focus on analyzing the advantages and limitations of both traditional and emerging techniques. Additionally, it explores the potential of multimodal fusion strategies and artificial intelligence (AI) in EPD. At present, machine vision, wearable monitoring, and several AI applications remain prospective approaches rather than validated tools for routine EPD. The conclusion highlights that, despite significant progress, current technologies still face limitations in achieving in situ, non-contact, and high-throughput detection. Looking ahead, the integration of cutting-edge technologies, such as AI, small wearable sensors, and physiological time-series data analysis, holds promise for overcoming these bottlenecks, enabling more intelligent and efficient pregnancy diagnosis, and providing scientific support for modern animal husbandry.

## Simple Summary

Early identification of pregnancy enables farmers to separate pregnant and non-pregnant animals, adjust feeding, repeat breeding sooner, and reduce unnecessary handling. This review summarizes the development of early pregnancy diagnosis in livestock, from observation and palpation to hormone and pregnancy-associated protein tests, ultrasound, thermal imaging, optical sensing, wearable devices, and computer-assisted analysis. Conventional methods are inexpensive and practical but can be operator-dependent or reliable only after a certain stage of pregnancy. Laboratory tests and ultrasound generally provide higher accuracy, although they require sampling, equipment, or trained personnel. Emerging non-invasive sensors can collect information rapidly with less disturbance to animals, but their performance varies among species, farms, devices, hair conditions, and pregnancy stages. Combining complementary information, such as ultrasound, molecular markers, temperature, and optical signals, may improve reliability, but larger and independently validated studies are still needed. No single method is optimal for every livestock production system. Future tools should be accurate, affordable, easy to use on farms, and validated under diverse practical conditions.

## 1. Introduction

In modern large-scale animal husbandry, reproductive management stands as the cornerstone for enhancing production efficiency and economic viability [1,2,3,4]. Efficient reproductive management not only ensures the optimal utilization of resources but also significantly contributes to the overall profitability of livestock operations [5,6]. Among the various aspects of reproductive management, rapid and accurate EPD in livestock plays a pivotal role. The ability to identify pregnancy early and accurately is crucial for optimizing breeding cycles and supporting sustainable livestock production. This process directly impacts reproductive efficiency. Timely detection of pregnancy allows for targeted care of pregnant animals, ensuring they receive the appropriate nutrition and healthcare. Simultaneously, non-pregnant animals can be promptly subjected to estrus management and artificial insemination, facilitating the initiation of a new breeding cycle.

EPD refers to the use of scientific methods to determine pregnancy status during the early post-breeding period. The meaning of “early” depends on both embryo-maternal signaling and production-management requirements. Maternal recognition signals, such as interferon tau, become prominent in cattle and sheep approximately 15–19 days after conception, and insufficient signaling may be followed by luteolysis and pregnancy failure [7,8]. Conceptus elongation and initial attachment also occur at species-specific times, including approximately days 19–22 in cattle and days 15–18 in pigs [9].

For the purposes of this review, “very early” refers to pregnancy assessment conducted before or around maternal recognition of pregnancy, whereas “early” refers to assessment conducted after maternal recognition but before the species-appropriate window for conventional confirmatory diagnosis. Because reproductive timing differs among livestock species, no universal post-breeding day cutoff is applied. Each method is therefore described together with the target species and the actual post-breeding interval at which it was evaluated. We also clarify whether the reported finding represents the detection of a pregnancy-associated signal, a presumptive screening result, or a confirmatory diagnosis. Because pregnancy generally becomes easier to determine as gestation progresses, diagnostic performance reported at substantially different post-breeding intervals is not treated as directly comparable.

Timely pregnancy diagnosis can support stage-appropriate feeding because energy and nutrient requirements vary with physiological status and gestational stage in both sows and cattle [10,11]. It can also facilitate the prompt reproductive examination and rebreeding of nonpregnant females. For example, milk PAG testing on day 28 has been associated with fewer days open than later rectal pregnancy diagnosis [12]. Reproductive surveillance may also prompt follow-up after pregnancy loss and support the separate evaluation of postpartum uterine disorders that impair fertility. Pregnancy testing itself, however, does not diagnose or prevent endometritis [13,14,15].

However, the global livestock sector currently faces rising labor costs and increasing pressure to adopt intelligent management systems. Traditional EPD methods, such as rectal palpation, depend heavily on operator experience and may therefore produce variable results. Hormone testing has method-specific limitations related to both timing and pregnancy specificity. Laboratory-based assays may involve delays in sample processing and result reporting, and the earliest reliable sampling window varies among species and assays. Moreover, an elevated P4 concentration is not pregnancy-specific because it reflects sustained luteal activity and does not by itself confirm pregnancy [4]. These method-specific limitations, together with the operational constraints of conventional methods, have motivated the development of EPD technologies that are efficient, cost-effective, and practical for livestock production.

Recent reviews have provided important but complementary perspectives on livestock pregnancy diagnosis. Szenci [16] focused on field diagnosis of early pregnancy and pregnancy loss in dairy cows, whereas Ott et al. [9] synthesized pregnancy-establishment biology and the principal diagnostic methods used in cattle, sheep, goats, pigs, horses, and camelids. Together, these reviews establish the biological and clinical foundations of EPD. However, a cross-technology comparison is still needed to evaluate established and emerging methods within a common framework encompassing species-specific testing windows, diagnostic purpose, pregnancy reference standard, performance metrics, validation design, operational requirements, and readiness for on-farm use.

The present review addresses this need by comparing conventional examination, P4 and PAG assays, molecular markers, B-mode and Doppler ultrasonography, IRT, optical sensing, wearable devices, machine vision, AI, and multimodal fusion across these dimensions. It also extends the species coverage to buffaloes and rabbits, which were not central to the two previous syntheses. Importantly, this review distinguishes analytical detectability from validated diagnostic performance, presumptive screening from confirmatory diagnosis, and internal or cross-batch evaluation from independent external validation. Its contribution is therefore not simply an updated catalog of techniques but a critical assessment of the maturity of the evidence, practical constraints, and readiness of EPD methods for translation to livestock production.

## 2. Review Methodology

To improve transparency and reproducibility, the literature search and selection process was documented using predefined eligibility criteria, database searches, and staged screening.

### 2.1. Review Design and Eligibility Criteria

**Figure 1:**
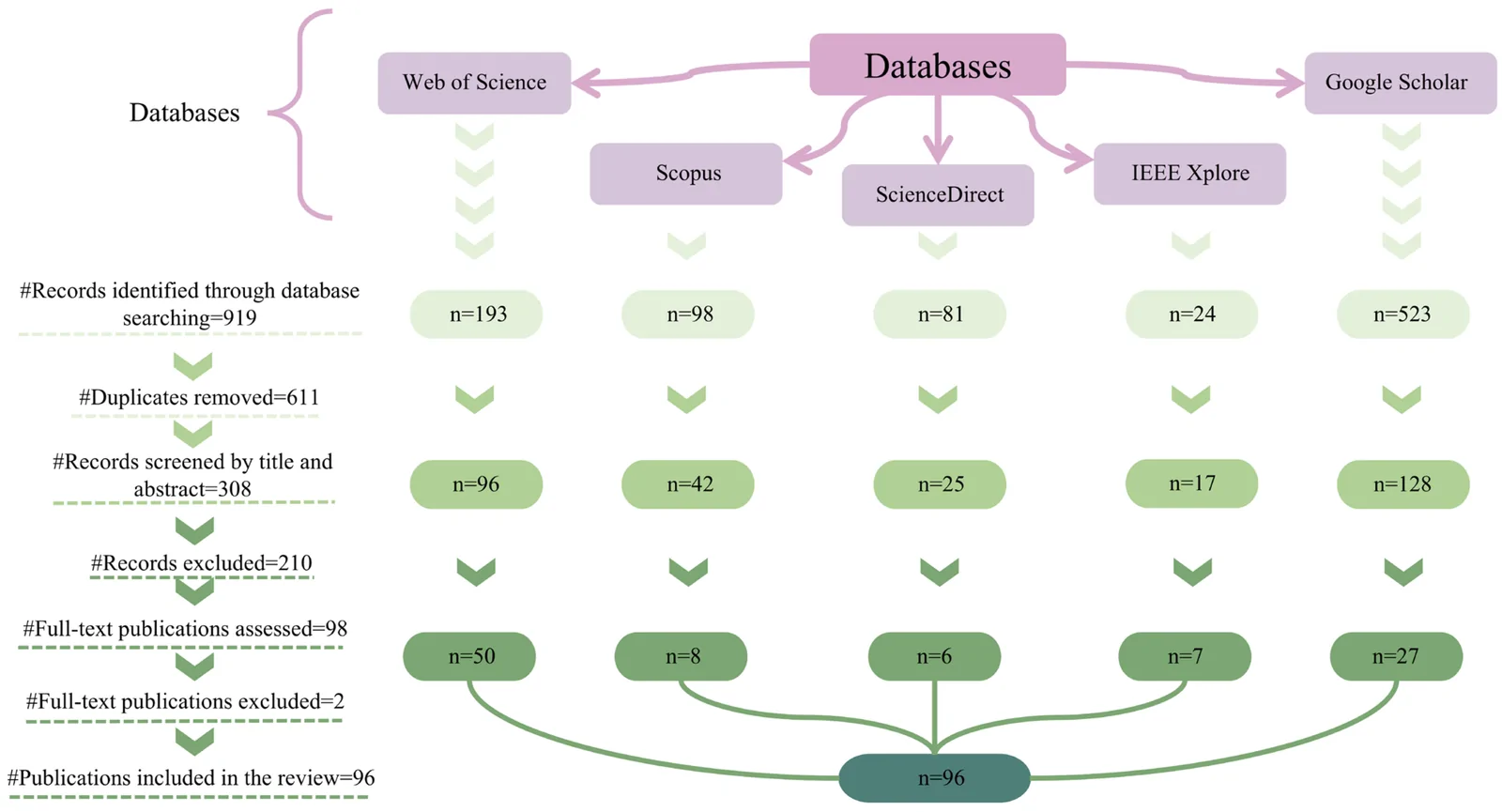
Literature identification and selection process.

To provide a comprehensive review of the development and application of early pregnancy diagnosis (EPD) technologies in livestock, this review considered studies published through 31 July 2026. Primary evidence was drawn from English-language, peer-reviewed journal articles. One historical monograph and one authoritative reference chapter were used only to provide historical or technical context. These non-primary sources were not included in the journal-article selection count shown in Figure 1. Conference papers were excluded. The eligibility criteria were as follows:

1. Primary evidence sources were English-language, peer-reviewed journal articles that directly investigated pregnancy detection, screening, diagnostic validation, or the technical application of EPD-related methods in livestock.

2. The included studies were required to be relevant to livestock EPD and to address different technical methods and their potential or practical applications in livestock production.

3. The included publications were required to report relevant technical details or experimental findings concerning the investigated method.

4. The studies were required to provide information on at least one of the principal dimensions evaluated in this review, including the applicable livestock species, post-breeding assessment interval, diagnostic purpose, pregnancy reference standard, diagnostic performance, operational requirements, or validation design.

5. For historical sources, we included the earliest original studies reporting relevant methods or pregnancy-associated signals, as well as authoritative monographs documenting their discovery, standardization, or early application in livestock. These sources were used primarily to trace the historical development of livestock EPD technologies.

6. Studies of machine vision or related technologies that did not directly assess pregnancy were included in the Future Prospects section when they offered relevant technical guidance for EPD. These studies were not treated as direct evidence of diagnostic performance. Authoritative reference chapters used solely to provide technical context were treated as non-primary sources and were not included in the journal-article count shown in Figure 1.

### 2.2. Information Sources and Search Strategy

Relevant studies were searched in Web of Science, Scopus, ScienceDirect, IEEE Xplore, and Google Scholar, with the final search conducted on 31 August 2026. Three search sets were developed for livestock EPD, machine vision and AI, and wearable or multimodal sensing technologies. The search sets and database-specific field restrictions are summarized in Table 1.

### 2.3. Study Selection and Data Handling

All retrieved records were imported into Zotero for reference management and duplicate removal, followed by manual verification. Two authors independently screened the titles and abstracts and subsequently assessed the potentially eligible full-text articles. Disagreements were resolved through discussion, with a third author consulted when consensus could not be reached. Data on species, sample size, diagnostic timing, reference standard, performance metrics, and validation design were extracted from the included studies. When several studies examined the same technology, priority was given to studies with direct relevance to EPD, clearly reported methods, appropriate reference standards, and more complete diagnostic-performance data. The selection process is summarized in Figure 1.

## 3. Evolution of Early Pregnancy Diagnosis Technologies

### 3.1. Historical Development

The technology for EPD in livestock has evolved over more than a century, gradually transitioning from empirical approaches to precision techniques. Prior to the emergence of systematic medical documentation, herders and veterinarians primarily depended on empirical observations and palpation to ascertain whether livestock were pregnant. Empirical observation centered on detecting changes in the animals’ behavior and physical traits, while palpation entailed manually examining the abdomen or rectum to make a preliminary determination of the presence of a fetus or gestational sac in the uterus. Nevertheless, these methods were entirely contingent on individual experience and were susceptible to factors such as animal breed and rearing environment.

In 1956, the first edition of Stephen J. Roberts’s Veterinary Obstetrics and Genital Diseases provided a systematic clinical description of rectal palpation for pregnancy diagnosis, including the evaluation of characteristic uterine changes [17]. In 1964, X-rays were employed for the first time in pregnancy diagnosis of sheep, achieving a 96% accuracy rate by identifying fetal bones. This method enabled the examination of 400 to 600 sheep per day [18]. It was characterized by its speed and intuitiveness and could eliminate false pregnancies. However, due to the bulky nature of the equipment, concerns regarding radiation safety, and its inability to differentiate between normal amniotic fluid and pathological fluid accumulation, this method was gradually phased out.

The 1960s witnessed the emergence of ultrasound technology. A-mode ultrasound devices, with their low accuracy and high false positive rate, were not widely adopted [19]. Doppler ultrasonography was subsequently investigated for pregnancy-related screening, although the applicable post-breeding interval and diagnostic performance varied among species and study protocols. In 1976, B-mode ultrasound was applied to pregnancy diagnosis in ewes. The scanner used in that study had previously been developed to measure backfat and loin-eye area in pigs [20]. Compared to A-mode ultrasound, B-mode ultrasound enables real-time visualization of the number and vitality of fetuses within the uterus. Owing to its non-invasive and repeatable nature, B-mode ultrasound has gradually replaced X-ray technology.

In 1978, Thirapatsukun et al. reported a significant increase in plasma progesterone (P4) in pregnant females [21]. Plasma P4 can be measured by radioimmunoassay (RIA). However, an elevated P4 concentration is not pregnancy-specific because luteal cysts or delayed ovulation may produce false-positive classifications. Interpretation therefore requires consideration of the estrous cycle. In 1979, EPF was evaluated as an early pregnancy marker in livestock. Morton et al. detected EPF in ewe serum within 72 h of mating using the RIT [22]. This study demonstrated the early detectability of EPF in ewe serum, but its diagnostic sensitivity and specificity were not established and require further validation. In 1992, bPAG was detected in the serum of pregnant cattle using RIA. The cited study reported that the assay could support pregnancy diagnosis in cattle from approximately day 28 after breeding, with diagnostic sensitivity exceeding 90% [23]. Subsequent ELISA development improved assay throughput and supported the use of PAG testing as a screening approach. In the 2010s, circulating miRNAs were investigated as pregnancy-associated signals in cattle and buffaloes. In 11 Holstein–Friesian heifers, miR-26a levels increased as early as day 8 after insemination [24]. Guelfi et al. also reported differential serum miRNA expression between pregnant and nonpregnant buffalo cows at day 40 [25].

In the 2020s, infrared thermography and optical sensing emerged as non-invasive approaches to EPD. Lai et al. [26] developed a wearable matrix optical device incorporating LEDs at 660, 850, and 940 nm to measure pregnancy-related differences in abdominal optical signals in rabbits. In buffaloes, Riaz et al. [3] reported significant region-specific surface-temperature differences between pregnant and non-pregnant animals, but did not report diagnostic sensitivity or specificity. Olğaç et al. [27] likewise identified potentially informative thermographic differences in Holstein cows and heifers. Together, these studies demonstrate feasibility while also indicating the need for larger and externally validated diagnostic studies.

**Figure 2:**
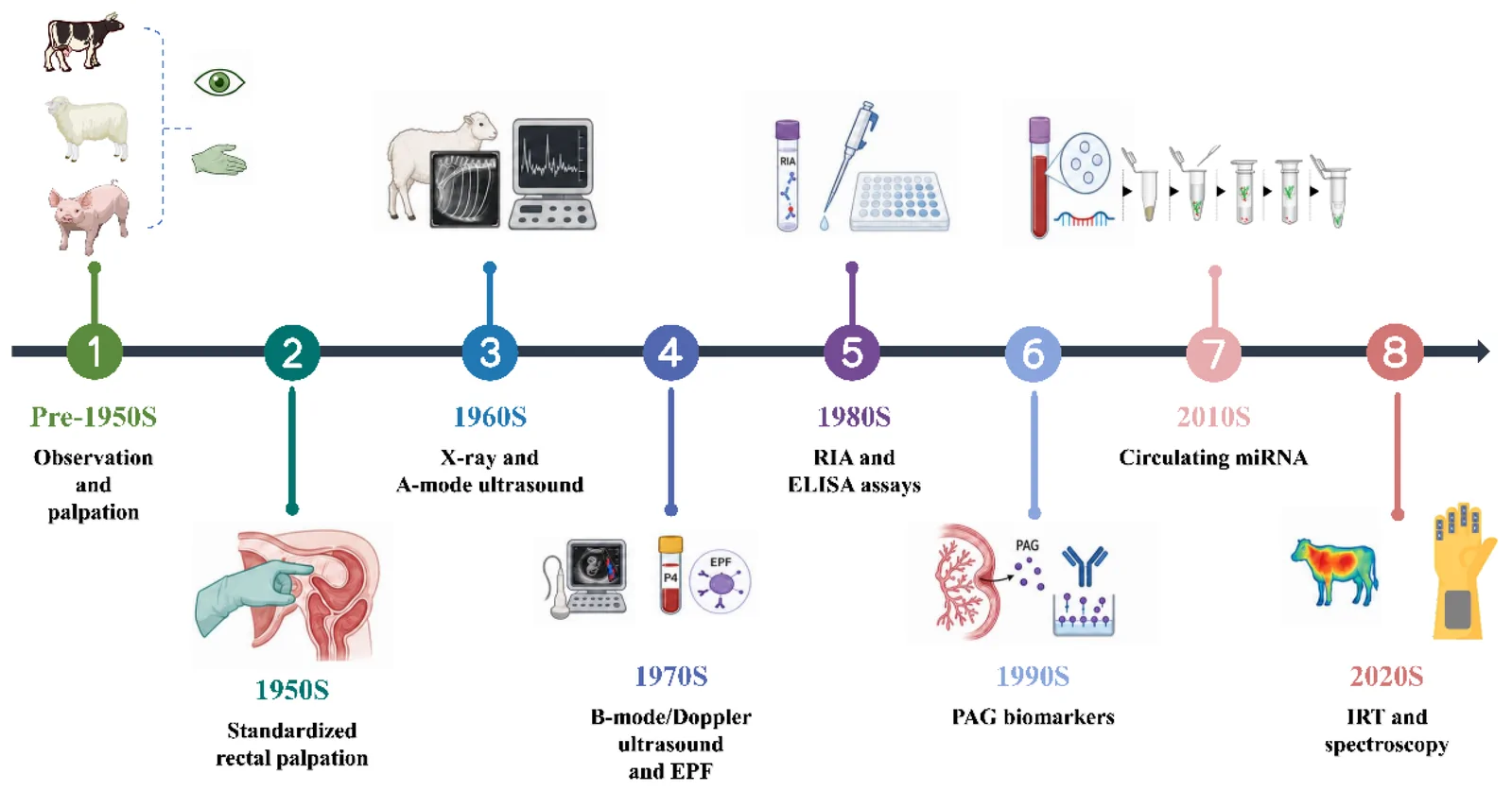
The evolution of EPD techniques in livestock.

As shown in Figure 2, EPD technologies have progressed from empirical physical examination to molecular, imaging, and sensor-based approaches. However, these methods differ substantially in applicable timing, diagnostic purpose, and level of validation. Established diagnostic methods should therefore be distinguished from pregnancy-associated signal detection and exploratory screening approaches.

### 3.2. Traditional and Laboratory-Based Methods

#### 3.2.1. Traditional Pregnancy Diagnosis Methods Based on Physiological Changes

Traditional pregnancy diagnosis methods rely mainly on observing physiological and behavioral characteristics in livestock. Commonly used techniques include rectal palpation, abdominal palpation, and direct behavioral observation. These methods may provide preliminary pregnancy information, but their applicable timing varies substantially among species. In some livestock, palpation becomes reliable only at relatively late gestational stages. The suitability of a method depends on the species, days post-breeding, equipment availability, and required diagnostic accuracy.

Rectal palpation is widely used for pregnancy diagnosis in large ruminants and involves assessing the uterus through transrectal examination. The method is economical and requires no specialized equipment, but its accuracy depends substantially on operator experience. Karen et al. [28] reported a sensitivity of 93.8% for pregnancy detection by transrectal palpation in Egyptian buffaloes examined between days 46 and 50 of gestation. Recent methodological improvements, such as the standardized “bimanual combined” examination proposed by Bello et al. [29], may help improve examination consistency and reduce operator-related errors.

Abdominal palpation is used mainly as a late-pregnancy screening technique in rabbits and sheep. In Merino ewes examined at 90–130 days of gestation, abdominal palpation achieved an accuracy of 80–95%, and withholding feed and water for 12–24 h improved the ease of examination [30]. In rabbits, abdominal palpation can reliably diagnose pregnancy approximately 10–14 days after mating [31]. Accuracy depends on gestational stage, body condition, restraint, and operator experience, and improper handling may cause stress or injury.

Direct observation uses post-breeding changes in animal behavior and body conformation as indirect indicators of pregnancy. These changes may include reduced activity, increased resting behavior and appetite, weight gain, a calmer temperament, and increased abdominal circumference. This approach is simple, economical, and easy to implement. However, these signs are nonspecific and may be influenced by seasonal conditions, individual variation, and health status, which can increase the risk of misclassification.

Overall, traditional methods are advantageous for small-scale farming due to simplicity and low cost, but their accuracy and reliability heavily rely on operator skill and experience, limiting widespread and precise application in larger populations.

#### 3.2.2. Laboratory Testing Based on Biochemical and Molecular Markers

Compared with physiological observation, laboratory testing methods can provide more objective pregnancy-associated signals [32]. Since the 20th century, rapid advances in biochemistry and immunology have led to increasingly diverse laboratory pregnancy-testing technologies targeting molecules, proteins, and hormones.

##### Hormone Level Testing

**Figure 3:**
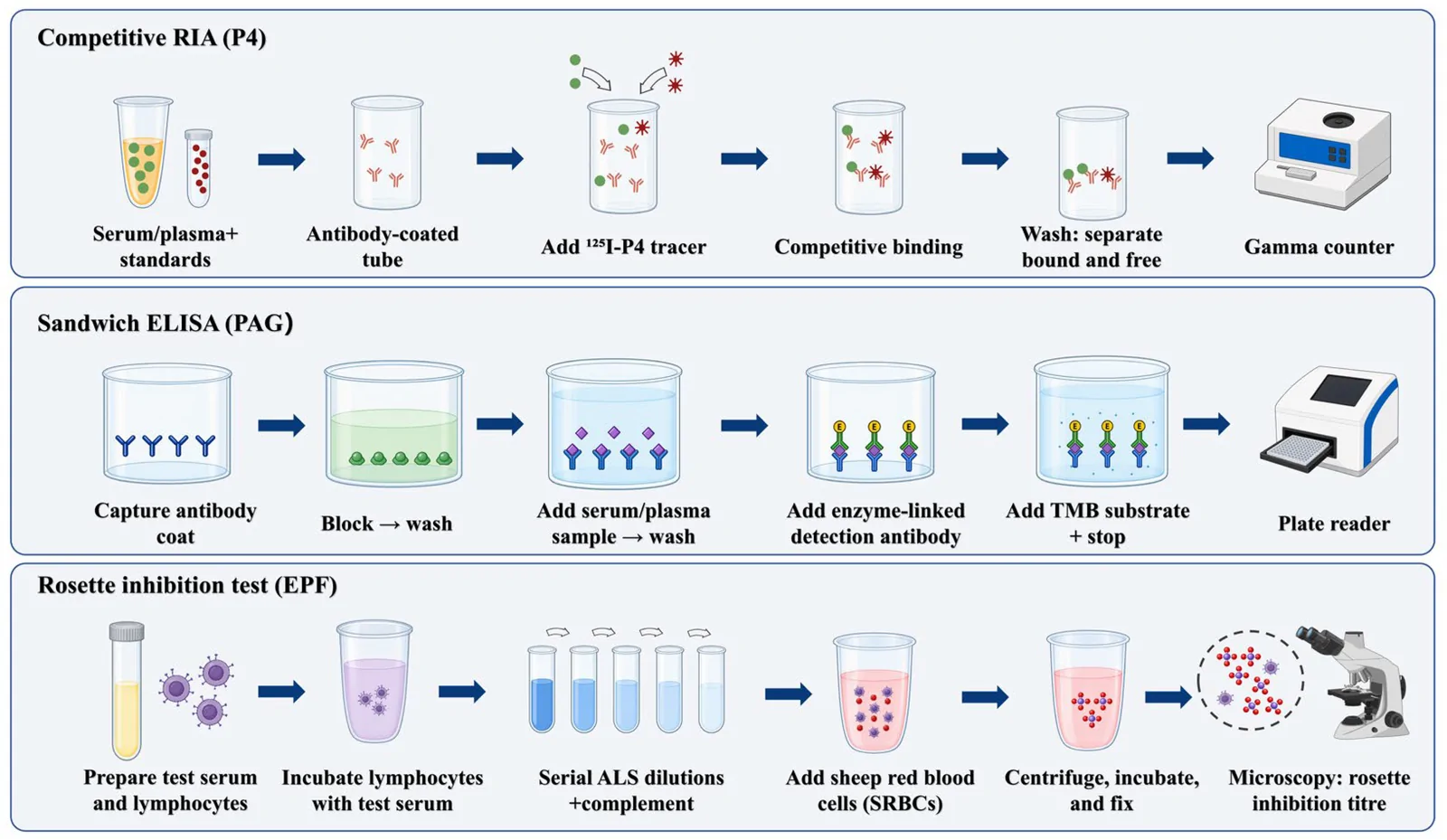
Detection flow chart of RIA, ELISA and RIT.

Progesterone (P4) is a naturally occurring progestogen secreted primarily by the ovarian corpus luteum and is essential for establishing and maintaining pregnancy. The term progestin generally refers to synthetic compounds with progestational activity. Following successful maternal recognition of pregnancy, luteolysis is prevented and luteal P4 remains elevated relative to animals that undergo normal luteal regression. Consequently, P4 concentrations in serum, plasma, or milk can be used mainly to identify non-pregnant animals, although elevated P4 alone does not confirm the presence of a viable conceptus. Common detection methods include radioimmunoassay (RIA) and enzyme-linked immunosorbent assay (ELISA), whose assay workflows are shown in Figure 3.

Because luteal physiology differs among species, both the diagnostic performance and the optimal sampling window of P4-based testing vary. In sows tested between days 17 and 22 after insemination, blood-spot P4 RIA correctly classified 98.8% of pregnant cases and 80.0% of nonpregnant cases, with an overall accuracy of 96.8% [33]. In ewes tested on day 18 after artificial insemination, P4 testing yielded a sensitivity of 100%, a specificity of 95.4%, a positive predictive value of 81.6%, and a negative predictive value of 100% [34]. In lactating Holstein cows, an in-line milk P4 system maintained sensitivity above 95% between days 27 and 54 after artificial insemination. However, specificity was below 90% before day 40 and exceeded 94% after day 41. Overall accuracy above 95% was achieved only when pregnancy was declared no earlier than day 41 after artificial insemination [35]. In Iraqi riverine buffaloes, P4 testing yielded a sensitivity of 66.7% and a specificity of 53.9% between days 22 and 24 after mating. Both sensitivity and specificity reached 100% between days 42 and 44 in the study population [36]. These findings demonstrate that the performance of P4-based testing depends strongly on species and sampling time, and that sensitivity, specificity, and overall accuracy should be reported separately.

P4 testing should be interpreted as a species- and time-specific screening method that is particularly useful for identifying nonpregnant animals. Positive classifications may require confirmation using PAG testing or ultrasonography at an appropriate post-breeding interval.

##### Early Pregnancy Factor Detection

In 1974, Morton et al. first reported pregnancy-associated immunosuppressive activity in pregnant mice using the RIT, laying the foundation for subsequent research on EPF [37]. The prevailing view among most studies is that EPF represents an immune-suppressive protein associated with pregnancy. It emerges in the blood and milk during maternal pregnancy, serving to prevent maternal immune rejection and ensure the survival of the fetus [38]. The commonly used detection method for EPF is RIT, and the detection process of RIT is shown in Figure 3.

EPF-related activity has been reported shortly after fertilization. In horses, EPF activity has been detected in serum within 24–72 h after fertilization, while early RIT responses have also been reported in cattle and pigs [38,39,40]. In dairy cows, RIT values measured 1–3 and 5–7 days after insemination differed significantly between inseminated animals and non-inseminated controls, indicating potential for first-week pregnancy screening [39]. However, these studies used small, species-specific samples and heterogeneous procedures, and they did not establish robust, externally validated estimates of diagnostic sensitivity and specificity. Therefore, EPF-based RIT should be regarded as an early experimental indicator rather than a fully validated field test.

EPF-related activity may become detectable earlier in gestation than pregnancy-associated changes identified by P4 assays or ultrasonography, suggesting potential value for investigating very early pregnancy. Experimental studies in sheep and cattle have reported declines in EPF activity following induced embryonic death or embryo removal. However, its diagnostic performance for detecting naturally occurring early embryonic loss requires further validation. EPF should therefore be regarded as a potential research indicator of embryo viability [41,42]. The conventional RIT requires an in vitro culture reaction system and the preparation of anti-lymphocyte serum. These requirements render the test technically challenging and time-consuming. Consequently, the technology is limited to processing a small number of samples at a time, making it unsuitable for large-scale screening. Although ELISA-based immunodiagnostic kits are available, they rely on expensive antibodies and reagent support, resulting in high experimental costs. More recently, a monoclonal antibody against synthesized porcine EPF was evaluated using sow urine collected on day 20 post-mating, but the preliminary diagnostic accuracy was 70%, underscoring the need for further validation before field use [43].

##### Detection of Pregnancy-Associated Glycoproteins

Pregnancy-associated glycoproteins (PAGs) are important protein biomarkers widely used for EPD in ruminants [44]. ELISA is the principal method used for PAG detection because it offers high analytical sensitivity and a relatively simple workflow.

PAGs are secreted by placental trophoblast cells in ruminants and enter the maternal circulation after placentation begins. Blood PAG concentrations typically reach detectable levels 21–28 days after mating. PAG concentrations in milk are approximately one tenth of those in blood. Accordingly, milk-based testing is generally performed 3–5 days later to achieve comparable diagnostic accuracy [45,46]. Placental PAG expression and optimal detection windows differ among species. In goats, placental PAG expression occurs relatively early, and plasma RIA has achieved diagnostic accuracy above 95% by day 21 after mating [47]. In buffaloes, the optimal detection window is later, generally 25–28 days after mating. Reported diagnostic accuracy in buffaloes has exceeded 98% [46,48]. Diagnostic sensitivity also varies with the assay format used within a species. For example, López-Gatius et al. [49] evaluated a commercial rapid PAG test and found that within the detection window of 28 to 55 days, the sensitivity of the rapid test was 100% with plasma, compared with 75–76% with whole blood. This study suggests that, when field detection conditions permit, plasma may be preferable when centrifugation is feasible because it reduces false-negative results. Recent studies have extended PAG testing toward rapid and minimally invasive formats. A latex-based immunochromatographic assay evaluated in 559 Holstein cows enabled field-oriented PAG detection from day 28 after breeding [50], while LC-MS/MS identified caPAG2 in goat milk on day 20 post-mating, highlighting the potential of milk proteomics for non-invasive early diagnosis [51]. In Karya and Konya Merino ewes, serum PAG profiles were similar across breeds, and pregnant and non-pregnant animals became distinguishable from week 4, supporting gestational-stage-specific interpretation in sheep [52].

In large-scale dairy farms, a rapid milk PAG test can be conducted at 28 days post-mating to initially screen out non-pregnant cows. For high-value breeding stock or in research settings, blood samples can be collected for ELISA testing at 23 to 24 days post-mating to facilitate earlier decision-making. Additionally, a re-examination between 60 and 74 days post-mating is recommended to enhance the confirmation rate of viable pregnancies. Interpretation of milk PAG results should nevertheless account for animal- and production-level factors. An analysis of 2460 dairy cows found associations with breed, gestational age, and parity, and approximately 12% of cows classified as pregnant did not subsequently calve, supporting the need for confirmatory follow-up [53].

##### MicroRNA Detection

miRNAs are a class of non-coding RNAs that are approximately 21–25 nt in length. In the first trimester, complex molecular communication occurs between the mother and the embryo, and miRNAs play an important role in regulating the fertility of the endometrium, embryo implantation, and placental formation. These miRNAs are expressed not only in tissues but can also be released into the circulation through exosomes and other carriers, forming stable circulating miRNA profiles that may serve as candidate biomarkers for liquid biopsy [54].

Compared with PAG testing or B-mode ultrasonography, circulating miRNAs have been investigated as pregnancy-associated signals at earlier post-breeding intervals [54,55,56]. However, they have not yet been established as routine diagnostic tools. Several studies have demonstrated that significant differences in miRNA expression can be detected approximately three weeks after breeding in ruminants such as cattle, sheep, and goats. Furthermore, miRNA detection is applicable to a variety of body fluid samples. While plasma and serum samples remain the mainstream choices, exosome-based enrichment techniques significantly enhance the signal-to-noise ratio of miRNAs [57,58]. In buffaloes, plasma miRNA profiling identified candidates that differentiated pregnancy status by day 18 after artificial insemination, including miR-181a and miR-486; in sows, serum exosomal miR-192 showed potential for pregnancy screening on day 14 [59,60]. Additionally, research on milk-derived miRNAs is actively underway, which will facilitate non-invasive sampling in large-scale dairy farms. The sensitivity of diagnosis using a single miRNA is generally limited. For instance, miR-126-3p exhibits a sensitivity of approximately 73%. However, employing a combination of multiple miRNAs or integrating machine learning algorithms can elevate the diagnostic performance, as indicated by an area under the curve (AUC) exceeding 0.90 [57,61].

Overall, circulating miRNAs are promising candidate biomarkers for EPD in livestock. However, reported miRNA profiles and diagnostic performance may vary with species, breed, parity, age, gestational stage, sample type, and analytical method. Large, multicenter studies with standardized sampling, prespecified diagnostic thresholds, and external validation are therefore required before routine application [54]. The cost and technical requirements of RT-qPCR also currently limit large-scale on-farm use [62]. Future research should evaluate standardized multimarker panels and determine whether combining miRNA profiles with PAG measurements or wearable-sensor data improves externally validated diagnostic performance.

##### Emerging Transcriptomic and Metabolomic Biomarkers

Interferon-stimulated gene expression and metabolomics are expanding the molecular toolkit for EPD. In buffaloes, a duplex RT-qPCR assay targeting ISG15 and LGALS3BP detected pregnancy-associated expression changes around day 20 post-insemination, with validation AUCs of 0.95 and 0.90 [63]. In Hu sheep, untargeted plasma metabolomics combined with random-forest analysis identified a panel of candidate metabolites for early pregnancy classification at days 19 and 30 [64]. These approaches may support earlier detection than conventional PAG or ultrasound testing, but they require independent multicenter validation, standardized sampling windows, and assessment of breed, diet, and management effects before routine on-farm use.

### 3.3. Imaging and Non-Invasive Technologies

#### 3.3.1. Ultrasonographic Advances

With the continuous development of medical imaging technology, ultrasound examination has been widely proven to be a safe and efficient means for pregnancy diagnosis and the study of mammalian embryonic development, and it is gradually being promoted and applied in the pregnancy diagnosis of livestock. Compared to radiography and laparoscopy, ultrasound technology has the advantages of being non-invasive, highly safe, and capable of real-time imaging.

##### A-Mode Ultrasound Equipment

A-mode ultrasonography detects reflected echoes from the maternal uterus and displays echo amplitude over time to support pregnancy assessment. Its limited spatial resolution makes interpretation susceptible to interference from physiological conditions such as urinary bladder distension and endometrial edema, which may produce false-positive classifications. A-mode ultrasonography is now used mainly for preliminary field screening and has largely been superseded by B-mode ultrasonography because of the latter’s greater diagnostic accuracy and broader applicability.

##### B-Mode Ultrasound Equipment

B-mode ultrasound is a diagnostic technique based on the imaging of sound wave reflections. Its working principle utilizes the differences in the intensity of reflected echoes at the interfaces of different tissues to construct real-time two-dimensional grayscale images [32]. The intensity of the echo signals is represented by brightness: areas with no echo or weak echoes appear dark on the screen, while strong echo structures are displayed in bright white. B-mode ultrasound belongs to the category of real-time imaging technology, capable of continuous scanning to obtain dynamic images, thereby capturing physiological activities such as fetal heartbeat.

**Figure 4:**
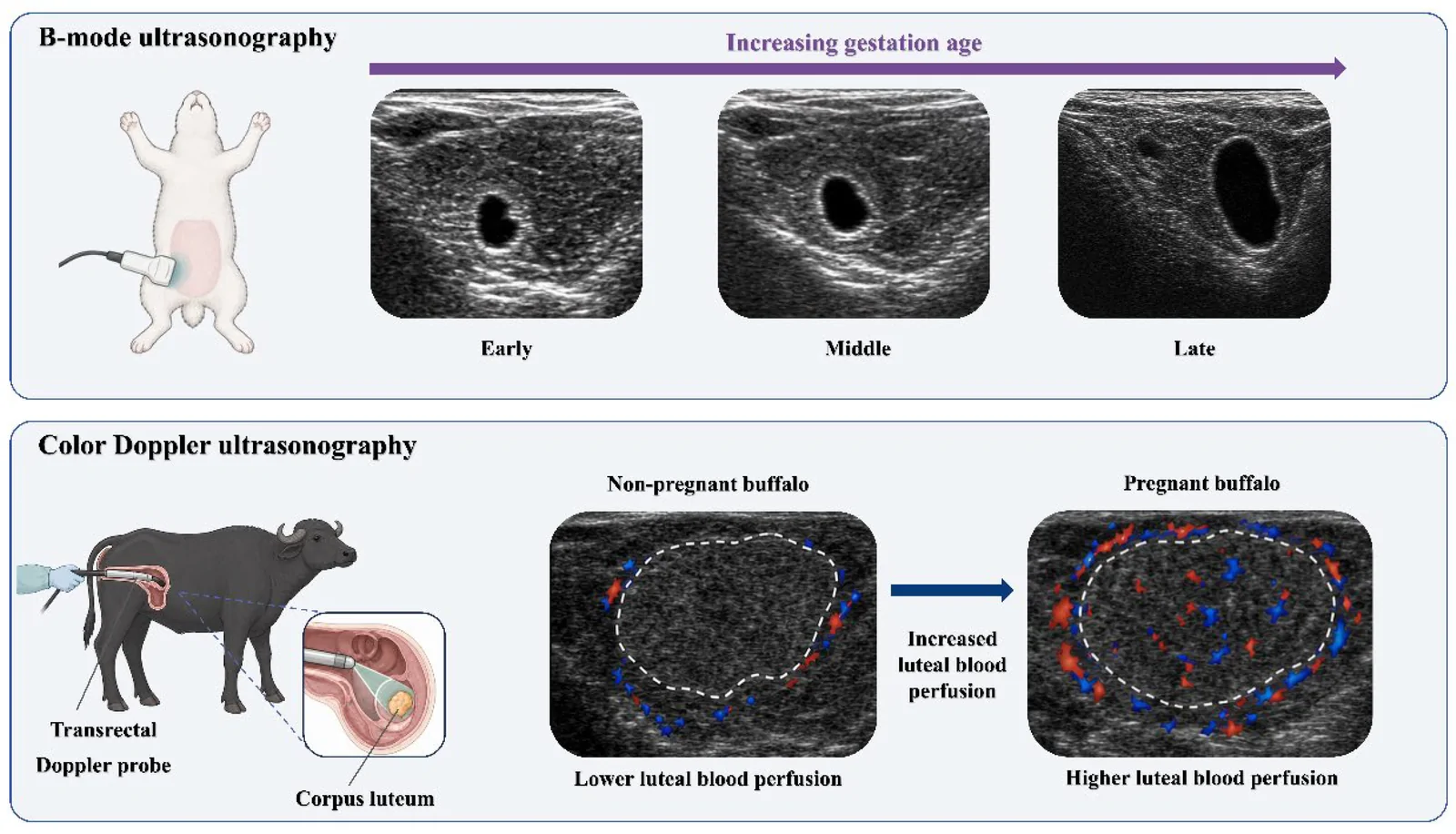
B-mode ultrasonographic visualization of rabbit gestational sac development and color Doppler assessment of luteal blood perfusion in buffaloes.

B-mode ultrasonography is an established diagnostic method for early pregnancy diagnosis in multiple livestock species and is commonly used as a reference standard when pregnancy-specific structures are visualized. It can also provide information on embryo number and viability. Owing to variations in uterine structure and embryonic developmental patterns, the earliest reliable detection window differs among species. In cattle, the relatively thick uterine wall and delayed embryo implantation result in a detection accuracy of 98% to 100% approximately 25 days post-breeding [32]. Goats and sheep exhibit similar timeframes to cattle [65]. Conversely, pigs and rabbits, characterized by thinner uterine walls, allow for earlier pregnancy diagnosis, with pigs detectable around day 18 and rabbits as early as days 7 to 9 post-breeding [66,67]. As embryonic volume increases and contrast between fluid-filled structures and surrounding tissues intensifies, the sensitivity of ultrasonographic diagnosis improves rapidly. As shown in the upper panel of Figure 4, the anechoic gestational sac progressively enlarges and becomes more clearly delineated on B-mode ultrasonography as gestation advances. For instance, in dairy cows, diagnostic sensitivity rises from 74.5% on day 24 to 100% by day 29 [68]. In sheep, sensitivity increases from 68% during the third to fourth weeks post-breeding to 100% thereafter [65]. Therefore, in practical production, it is essential to strike a balance between the timing of detection and the accuracy of results. A common approach involves conducting an initial examination between days 25 and 27 post-breeding in cattle, days 24 and 28 in sheep, days 25 and 30 in buffaloes, and days 20 and 21 in pigs, followed by a follow-up examination between days 40 and 60 to confirm litter size and embryonic survival.

B-mode ultrasonography, as a method for EPD, is widely acknowledged for its high accuracy. When performed within an appropriate time window and operated by experienced personnel, its diagnostic accuracy typically far exceeds that of traditional methods such as direct observation. Additionally, it provides extra embryonic information, making it an indispensable tool in modern breeding practices. More recently, AI-assisted ultrasonic biosensing has been used to suppress acoustic artifacts and annotate gestational structures in sow B-mode images. A YOLOv8-based system using animal-level separation demonstrated real-time image processing and improved early-gestation diagnostic performance, illustrating the value of embedded image enhancement while still requiring external validation [69].

##### Doppler Ultrasound Equipment

Doppler ultrasound emits ultrasound waves through a transducer and uses the Doppler effect to detect the motion state of the target tissue. When ultrasound encounters stationary tissues, the frequency of the reflected wave is consistent with that of the emitted wave. However, when it encounters moving organs or blood, the frequency of the reflected wave shifts (known as Doppler frequency shift) [70]. Some studies reflect the blood flow in the corpus luteum by calculating the percentage of the colored area on the cross-section of the corpus luteum relative to its area, or by having the examiner subjectively classify the blood supply of the corpus luteum into grades 1–4 based on the amount of color Doppler flow signals [71,72].

Recent studies indicate that color Doppler ultrasonography may support early pregnancy screening by assessing corpus luteum blood flow or uterine vascular signals. Figure 4 illustrates the differences in luteal blood perfusion between pregnant and nonpregnant cows [73]. Compared with B-mode ultrasonography, Doppler assessment may permit earlier pregnancy screening and provide indirect information related to luteal function. Studies have reported high sensitivity during this period because most pregnant animals retain detectable luteal perfusion, resulting in relatively few false-negative classifications [71,73,74]. However, high sensitivity should not be interpreted as high specificity. The diagnostic thresholds used to classify luteal perfusion have varied among species and studies. In ewes examined on day 17 after insemination, a subjective luteal vascularization score of 2 or greater yielded a sensitivity of 93.5% and a specificity of 80.8% [71]. In dairy cows examined on day 20, De Silva et al. [75] identified a study-specific cutoff of 51% for the incoming luteal blood-flow area, which yielded a sensitivity of 100% and a specificity of 85%.

Residual luteal perfusion may persist temporarily in nonpregnant females before complete luteolysis, producing false-positive classifications and reducing specificity. In postpartum beef cows, Holton et al. [76] reported that delaying color Doppler assessment from day 20 to day 22 increased specificity from 72% to 83% and overall accuracy from 87% to 92%, while sensitivity remained 100%. Therefore, a Doppler-positive result obtained at approximately day 20 should be regarded as presumptive rather than confirmatory. Applying validated species- and device-specific thresholds or delaying the assessment may improve specificity, but pregnancy should subsequently be confirmed by B-mode ultrasonography at an appropriate gestational stage. In dairy goats, color Doppler assessment of luteal blood flow permitted pregnancy prediction on day 21 after breeding, approximately two days earlier than B-mode assessment in the same study [77]. Computer vision has also been applied to luteal color Doppler videos to classify pregnancy status in beef cows. However, external validation is still required before routine application [78].

In cattle, sheep, buffaloes, and goats, Doppler ultrasonography may provide earlier pregnancy screening than conventional B-mode imaging. However, equipment cost, environmental susceptibility, the absence of standardized thresholds, and false-positive classifications associated with persistent luteal perfusion currently limit its use as a stand-alone diagnostic method. Reported thresholds may vary among species, breeds, ultrasound devices, and analytical procedures. Further research should therefore evaluate the temporal changes in luteal blood flow and establish externally validated, species-specific interpretation criteria.

#### 3.3.2. Infrared Thermography

IRT is a non-invasive monitoring technique that detects infrared radiation emitted from the animal surface and converts it into thermal images. It generally requires minimal animal handling and has been investigated as an exploratory approach for estrus monitoring and early pregnancy assessment in livestock.

During pregnancy, regional surface temperature may change in association with endocrine and hemodynamic adjustments. IRT therefore has potential as a non-contact screening method, but environmental conditions, anatomical site, animal age, and analytical thresholds must be controlled. In 27 Nili-Ravi buffaloes monitored from insemination to day 24, Riaz et al. [3] found higher selected surface-temperature measures at the left flank, left eye, and vulva in pregnant animals; the study was exploratory and did not report diagnostic sensitivity or specificity. In Holstein cows and heifers, Olğaç et al. [27] reported that the difference between anal and vulvar temperatures on selected post-insemination days could distinguish pregnancy groups, including 100% sensitivity at a threshold of 2.9 °C on day 6 in the study population. However, comparable performance was not observed consistently in heifers, underscoring the need for validation across age groups, environments, and herds.

In general, IRT performance is affected by ambient temperature, hair coverage, animal activity, measurement site, and instrument characteristics, and standardized interpretation criteria are not yet available. At present, IRT should therefore be regarded as an exploratory, non-contact screening approach rather than a stand-alone pregnancy diagnostic method. Measurements obtained immediately after breeding should not be used to make rebreeding decisions because the optimal testing window, diagnostic thresholds, sensitivity, and specificity have not been sufficiently validated. Any tentative pregnancy classification obtained by IRT should be confirmed using an established method, such as B-mode ultrasonography or an appropriately validated biochemical assay, at the relevant post-breeding interval before reproductive management decisions are made. Future studies should develop and externally validate decision models across breeds, ages, environments, and production systems.

#### 3.3.3. Non-Invasive Optical Sensors

In recent years, bio-detection technologies based on differences in tissue optical properties have garnered significant attention. The underlying principle involves wavelength-dependent absorption and scattering by tissue constituents, allowing spectral features to reflect biochemical composition and microstructure. Foundational optical studies have shown that intracellular structures and proliferative status influence light-scattering behavior [79]. This principle supports non-invasive tissue sensing, but pregnancy-specific discrimination must be established through direct studies in each target species.

Building on this principle, studies have progressively translated visible-near-infrared (Vis-NIR) diffuse reflectance spectroscopy from an exploratory approach into portable and wearable systems for pregnancy diagnosis in does. Yuan et al. [80] first proposed a non-invasive method for classifying pregnancy status in does using spatially resolved diffuse reflectance spectral features. Spectral data collected from abdominal tissues were used to develop and compare partial least squares discriminant analysis (PLS-DA) and support vector machine (SVM) models. The SPA-SVM model yielded a sensitivity of 93.18%, a specificity of 94.44%, and an accuracy of 93.88% in the validation set. The corresponding values in the prediction set were 86.96%, 90.00%, and 90.69%, respectively. Because model development and evaluation were conducted within the same study population, these results represent internal validation rather than independent external validation [80].

Subsequently, Yuan et al. [81] developed a wearable multispectral device to improve portability and facilitate practical application. The sensing probe incorporated three LEDs with wavelengths of 660, 850, and 930 nm and two silicon photodiodes. Data from 419 does were used for model development, and an additional 218 does formed a separate prediction cohort. In the hair-removed subgroup of this prediction cohort, the SVM model achieved an accuracy of 83.94%, a recall of 83.67%, a precision of 81.19%, and an F-score of 82.41%. The use of additional does for prediction provided animal-level separation between the model-development and final prediction cohorts. However, because the evaluation remained within the same overall research setting, it should not be considered independent multi-site external validation [81].

**Figure 5:**
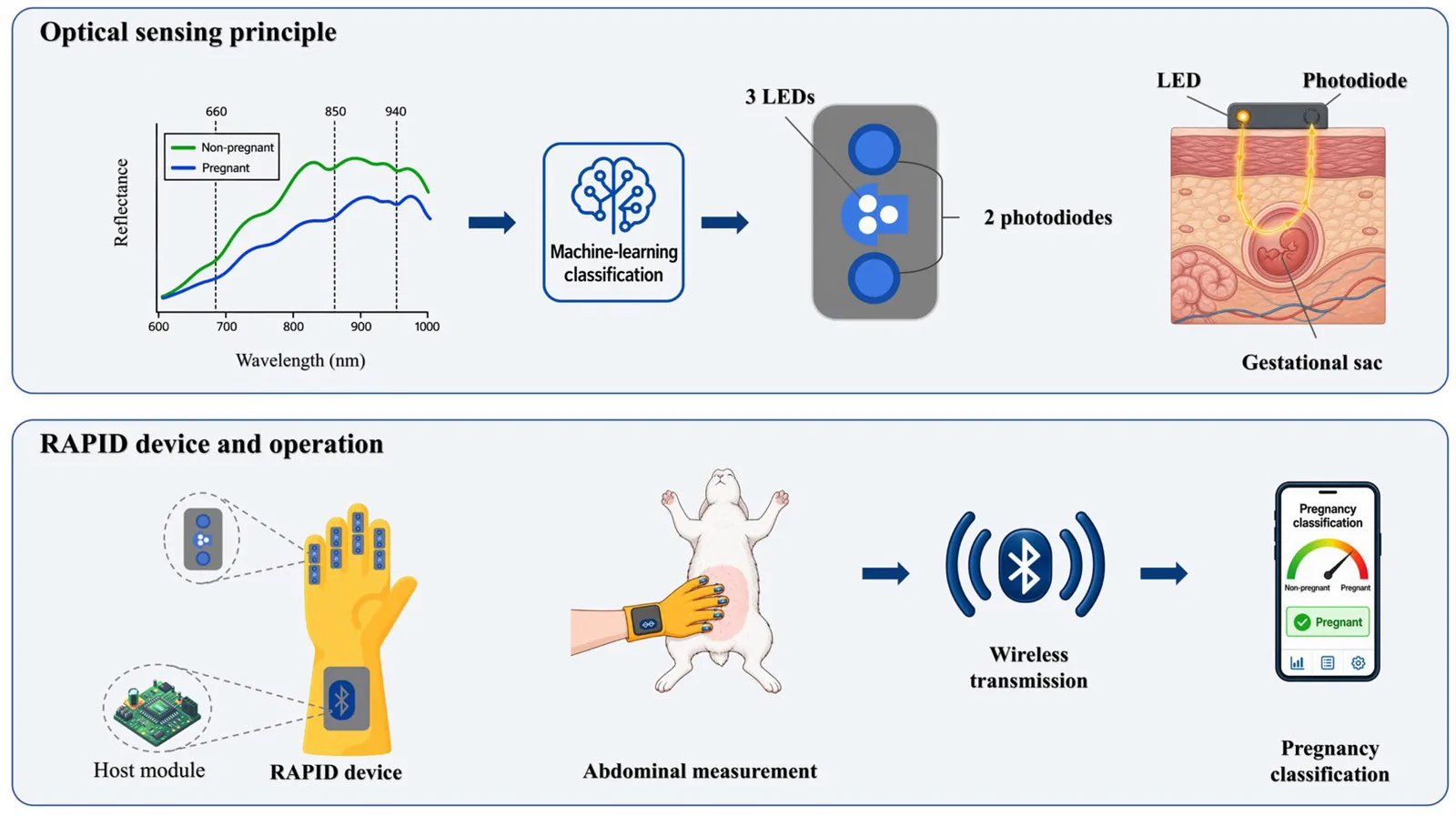
Schematic diagram of RAPID wearing and operation.

Building on the multispectral approach, Lai et al. [26] developed a wearable matrix optical sensing device known as RAPID. As shown in Figure 5, RAPID integrates eight sensor modules, each containing LEDs with wavelengths of 660, 850, and 940 nm and two photodetectors, enabling synchronous data acquisition from multiple abdominal regions. The device communicates with a mobile terminal through Bluetooth for device control and data visualization. In the primary analysis of data collected from 216 does, a Medium Gaussian SVM model achieved an accuracy of 86.63% and a recall of 80.49% on day 14 after insemination. When the model was evaluated using another batch of samples, its accuracy decreased to 78.36%. This cross-batch decrease indicates sensitivity to dataset shifts associated with age, abdominal hair density, and batch-specific conditions. The result should therefore be interpreted as cross-batch evaluation rather than full external validation [26].

Compared with ultrasonography or laboratory assays, optical sensing does not require invasive sample collection and can be integrated into lightweight, portable devices. The studies reviewed above also illustrate the progressive development of this field from exploratory spectroscopic measurements to portable and matrix-based optical sensing systems. However, internal validation, independent-animal holdout evaluation, cross-batch evaluation, and external validation should be clearly distinguished. When multiple measurements are obtained from different anatomical sites or through repeated examinations of the same animal, all observations from that animal should be assigned exclusively to either the model-development dataset or the evaluation dataset to prevent information leakage. Ultimately, the practical value of non-invasive optical devices will depend on whether their models are adequately validated across independent farms, breeds, age groups, devices, and operators.

### 3.4. Emerging Multimodal Fusion Approaches

Multimodal fusion is an emerging research approach that integrates two or more physiological, biochemical, or imaging signals to assess pregnancy status. In principle, combining complementary measurements may improve pregnancy classification when the individual modalities have different sources of error.

At 19–20 days after artificial insemination, Madoz et al. [82] jointly evaluated corpus luteum blood perfusion using color Doppler ultrasonography, plasma progesterone (P4) concentrations, and interferon-stimulated gene expression in peripheral leukocytes. In the subgroup with all three measurements available, the combined classification achieved 100% sensitivity for identifying cows considered possibly pregnant. Pregnancy status was subsequently determined by B-mode ultrasonography on days 33–34. However, pregnancy losses occurring between the two examinations complicate the interpretation of false-positive classifications and specificity. Other studies have similarly reported high sensitivity and negative predictive values for early Doppler assessment. Nevertheless, residual luteal perfusion before complete luteolysis may produce false-positive classifications and reduce specificity [75,76,83]. Multimodal assessment should therefore be regarded as a complementary screening approach rather than a replacement for confirmatory ultrasonography. It may provide useful information at approximately day 20 after insemination, whereas PAG testing is commonly applied from approximately day 28 and reliable B-mode ultrasonography is generally performed from approximately days 25–30 [68,84].

Several limitations remain. The molecular and biochemical components used in current multimodal studies largely rely on laboratory-based qPCR or ELISA. Rapid, portable, and validated testing platforms are therefore needed for integrated on-farm application. The available evidence remains preliminary, and multimodal fusion should not yet be considered an established or stand-alone diagnostic strategy. Most of the multimodal combinations reviewed here have been evaluated within a single research setting. Prospective external validation is therefore required before routine application.

## 4. Discussion

EPD methods differ in both their species applicability and the post-breeding intervals at which they can provide reliable results. Even for the same method, the optimal testing window may vary among livestock species. EPF-related activity and some molecular signals may be detected very early. For example, EPF was detected in ewe serum within 72 h of mating, and subsequent studies explored its association with embryo viability in cattle. However, these findings demonstrate analytical detectability rather than validated pregnancy classification [22,42]. P4 testing has been evaluated in sows, ewes, cattle, and buffaloes, but its appropriate sampling window and diagnostic performance differ markedly among species and gestational stages. Previous studies evaluated P4 between days 17 and 22 in sows, on day 18 in ewes, between days 27 and 54 in dairy cows, and between days 22 and 44 in buffaloes [33,34,35,36]. PAG assays generally become informative later than EPF-related activity and early luteal markers, but they have a more mature evidence base in ruminants, including cattle, sheep, and buffaloes. Their effective diagnostic window commonly begins during the third or fourth week after breeding, depending on the species, assay, and sample type [23,36,84,85]. Color Doppler ultrasonography has been investigated for earlier pregnancy screening in ewes, goats, cattle, and buffaloes. Nevertheless, residual luteal perfusion may persist temporarily in nonpregnant females before complete luteolysis. An early Doppler-positive result should therefore be regarded as presumptive rather than confirmatory [71,72,76,77]. B-mode ultrasonography becomes suitable for confirmation when pregnancy-specific structures, such as the gestational sac, embryo, or fetal heartbeat, can be visualized and has been applied in sheep, cattle, and pigs [20,68,86]. Consequently, diagnostic performance reported for different species, gestational stages, and intended uses should not be regarded as directly comparable. Table 2 provides a comparative summary of representative EPD methods, including their applicable species, diagnostic timing, reported performance, pregnancy reference standards, validation designs, advantages, and limitations.

Practical adoption also depends on invasiveness, cost, animal handling, and operator dependence. Rectal palpation has low equipment requirements and remains useful under field conditions, but it is invasive and highly dependent on examiner experience [32]. P4 and PAG assays provide more objective results. However, blood-based formats require venipuncture, while both blood- and milk-based formats require reagents and laboratory or validated on-farm analytical platforms [35,84]. B-mode ultrasonography allows direct visualization of pregnancy-specific structures but requires relatively expensive equipment and trained operators. Transrectal examination requires appropriate restraint and probe manipulation, whereas transabdominal examination may require animal positioning, hair clipping, and the application of acoustic coupling gel. These procedures increase preparation time and animal handling and may limit throughput in large herds or in smaller livestock species [68,86]. In contrast, IRT and optical sensing can acquire information without invasive sample collection. IRT has been investigated in cattle and buffaloes, but surface-temperature measurements are affected by ambient conditions, anatomical site, hair coverage, animal activity, and camera characteristics. The available pregnancy studies remain exploratory and have not established standardized decision thresholds across herds and environments [3]. Pregnancy-specific Vis-NIR and multispectral optical sensing in the studies reviewed here has been evaluated primarily in does. These methods are non-invasive and suitable for integration into portable devices, but their performance may be influenced by abdominal hair density, animal age, measurement position, device configuration, and batch-specific conditions [26,80,81]. IRT and optical sensing may therefore offer advantages for non-contact and potentially scalable monitoring, but their device calibration and model generalizability remain less established than those of conventional diagnostic methods.

The maturity of the available evidence should be interpreted according to the validation design. Internal validation evaluates model performance using data partitions or resampling procedures derived from the same study population. Independent-animal holdout evaluation prevents observations from the same animal from appearing in both model-development and evaluation datasets, but the animals may still originate from the same farm, device, operator, and acquisition period. Cross-batch evaluation provides a stronger assessment of temporal or batch-related dataset shifts. In contrast, external validation requires a prespecified and locked model or diagnostic threshold to be evaluated in an independent population and preferably at different farms or research centers. Among currently available methods, B-mode ultrasonography and appropriately validated PAG assays have the most established evidence for routine pregnancy diagnosis. P4 can support reproductive screening when species-specific sampling windows are applied, while Doppler ultrasonography provides complementary early screening information but still requires standardized interpretation thresholds and subsequent confirmation. IRT, optical sensing, wearable monitoring, multimodal approaches, and several machine-vision applications remain at different exploratory stages. Yuan et al. [80] reported internal model validation, whereas Yuan et al. [81] used an additional independent-animal prediction cohort within the same research setting. Lai et al. [26] subsequently reported a decrease in accuracy to 78.36% during cross-batch evaluation, illustrating the sensitivity of optical models to changes in animal characteristics and acquisition conditions. Pregnancy-specific machine vision has been evaluated using animal-level cross-validation in beef cattle and an independent cohort in sheep, but broader validation across farms, breeds, devices, and operators remains necessary [78,87]. Wearable-sensor and multimodal studies have likewise identified potentially informative physiological, behavioral, biochemical, and imaging signals, but most evidence has been generated within individual research settings [76,82,88]. Future studies should therefore use prospective protocols, independent animals, prespecified diagnostic thresholds, and multicenter external validation covering different farms, breeds, physiological stages, devices, environmental conditions, and operators before these emerging approaches are adopted routinely.

## 5. Future Prospects

With the continuous advancement of wearable sensors, deep learning-based image analysis techniques, and time series data analysis technologies, livestock pregnancy detection technology is poised to evolve toward in situ, non-contact, low-stress, and high-throughput approaches. These technological advancements will provide the animal husbandry industry with more intelligent and efficient management tools.

### 5.1. Application of Deep Learning and Image Analysis Technologies

#### 5.1.1. Semantic Segmentation as an Enabling Technology for Pregnancy-Related Imaging

Semantic segmentation is a computer vision technique that assigns an explicit semantic category to each pixel in an image, making it widely applicable to the automatic extraction of regions of interest in medical imaging. Compared with manual interpretation, deep-learning-based pixel-level segmentation can reduce observer subjectivity while improving consistency and automation. Common architectures include fully convolutional networks (FCNs) and various U-Net variants. U-Net is particularly widely used in biomedical image processing because of its strong feature-extraction capability and effective preservation of spatial information.

Semantic segmentation also has potential applications in livestock imaging. The automated segmentation algorithm developed by Xiberta et al. [89] achieved a mean F-score of ≥0.98 using CT data from nine live pigs, with a whole-body scanning time of approximately 50 s. Cao et al. [90] applied an Attention U-Net model to 1471 lumbar CT slices and reported an accuracy of 0.998, a mean intersection over union of 0.90, and a Dice coefficient of 0.95. These findings demonstrate that deep-learning models can address complex tissue boundaries and similarities in soft-tissue grayscale intensity [89,90].

In hyperspectral imaging, Seidlitz et al. [91] collected 506 hyperspectral images of porcine tissues and constructed a high-quality semantic segmentation dataset comprising 19 tissue classes. A U-Net model using whole-image input achieved a mean Dice coefficient of 0.90 and substantially outperformed segmentation based on RGB images [91]. These findings demonstrate the potential value of spectral information for improving tissue discrimination. In future studies, segmentation masks derived from hyperspectral images could potentially be registered with Doppler or thermal images, allowing blood-flow, temperature, and spectral features to be integrated within a common region of interest for comprehensive assessment.

Taken together, the CT and hyperspectral studies described above demonstrate the technical feasibility of automated tissue segmentation in livestock imaging. Because these studies did not directly evaluate pregnancy-specific outcomes, they provide an enabling technical basis for future research rather than direct evidence for EPD. Future studies should investigate whether similar network architectures can reliably segment pregnancy-specific structures, such as the uterine lumen, gestational sac, embryo, or corpus luteum, in livestock ultrasound images.

More recently, livestock-specific studies have begun to investigate pregnancy-related imaging more directly. Convolutional neural networks have been applied to luteal color Doppler videos for pregnancy classification in beef cows, while YOLOv8-based processing has been investigated for artifact suppression and annotation of pregnancy-related structures in B-mode ultrasound images from sows [69,78]. These studies represent pregnancy-specific exploratory applications rather than established diagnostic systems. Independent external validation is therefore required before their routine application.

#### 5.1.2. Machine Vision for General Phenotyping and Pregnancy Diagnosis

Machine vision refers to technological systems that combine imaging devices with computational algorithms to automatically acquire and interpret visual information for detection, measurement, classification, and process-control tasks.

For general phenotyping, machine-vision systems have been applied to both two-dimensional (2D) body-condition assessment and three-dimensional (3D) morphological measurement. Using LiDAR-derived point clouds and transfer learning, Huang et al. [92] achieved a point-cloud classification accuracy of 93.6%, while the error rates of the extracted linear body measurements were within 2.36%. Arrays of time-of-flight (ToF) depth cameras have also been used to reconstruct complete 3D meshes of cattle and derive body-surface area and volume measurements [93]. These studies demonstrate the ability of machine vision to quantify external livestock phenotypes automatically. However, they did not evaluate pregnancy status or establish a direct diagnostic relationship between external body morphology and EPD. Longitudinal monitoring of body-shape changes may provide complementary phenotypic information, but its diagnostic value for early pregnancy requires direct validation.

Machine vision has also been evaluated directly for pregnancy diagnosis using ultrasound images and videos. Gonçalves et al. [78] applied convolutional neural networks to luteal color Doppler videos obtained from 390 beef females on days 20 and 22 after artificial insemination. Pregnancy status was confirmed by conventional ultrasonography on day 29, and animal-level cross-validation was used to prevent images from the same animal from appearing in both the training and testing datasets. On day 22, the Xception model achieved an accuracy of 91%, a sensitivity of 99%, and a specificity of 79%. More recently, Golledge et al. [87] developed a convolutional neural network to classify pregnant and nonpregnant ewes from free-hand transcutaneous ultrasound videos. Evaluation in an independent cohort of 390 ewes yielded animal-level accuracies of 98.7% and 99.5% for the balanced and unbalanced models, respectively. These studies provide direct evidence that deep learning can support ultrasound-based pregnancy classification, although broader validation across farms, breeds, devices, and operators remains necessary.

External 2D and 3D imaging systems offer the advantages of non-contact data collection, high throughput, and minimal animal disturbance. Cameras and depth sensors can be installed in passageways or housing facilities to support automated and repeated phenotypic monitoring. Ultrasound-based machine vision still requires probe contact and appropriate animal handling. Nevertheless, deep-learning models can rapidly process ultrasound images or video frames, standardize image interpretation, reduce operator-related variability, and potentially support real-time diagnostic decision-making.

#### 5.1.3. AI Challenges in Livestock EPD

Deep learning and other AI methods may support livestock EPD, but their development and deployment remain constrained by data quality, generalizability, interpretability, and computational requirements. Large, well-annotated veterinary imaging datasets are scarce, and manual annotation is time-consuming and requires domain expertise. Insufficient training data can reduce model performance across gestational stages and imaging conditions [86]. Models trained on data from a single breed, farm, device, or operator may also perform inconsistently in new settings, underscoring the need for animal-level data separation and external validation across heterogeneous populations. Opaque model decision processes may limit clinical confidence, particularly when errors could affect reproductive management. Practical deployment may be further constrained by limited farm-side computing resources and the processing demands of ultrasound images and videos. Future work should therefore prioritize multicenter datasets, standardized annotation protocols, leakage-safe validation, interpretable outputs, and computationally efficient models suitable for edge deployment.

### 5.2. Application Potential of Small Wearable Devices

Compared with other pregnancy detection methods, small wearable devices can capture physiological changes and behavioral patterns in livestock in greater detail, potentially providing complementary technical support for EPD. Common wearable devices include neck collars, ear tags, and leg-mounted sensors. These devices typically integrate multiple sensors, such as accelerometers, gyroscopes, temperature sensors, and heart-rate monitors, and can continuously record activity, body temperature, heart rate, feeding, and rumination [94,95,96,97]. At present, these devices are used primarily for general health, estrus, welfare, and behavioral monitoring in precision livestock farming.

Pregnancy may be accompanied by changes in activity, rumination, feeding, or body temperature, and wearable sensors can capture these longitudinal signals. In dairy cattle, Gil et al. [98] reported transient increases in milk and body temperature between days 5 and 12 after artificial insemination in a high proportion of cows subsequently confirmed as pregnant. This finding suggests that temperature changes may contain pregnancy-related information. Early pregnancy may also be accompanied by changes in behavioral patterns, including reduced activity and changes in feeding and rumination duration. Wearable devices equipped with accelerometers and gyroscopes can continuously capture and analyze these subtle behavioral patterns [96,97]. Cavallini et al. [88] further reported differences in rumination and lying patterns between dairy cows subsequently classified as pregnant and nonpregnant after artificial insemination. However, these behavioral changes were proposed only as candidate indicators, and a validated pregnancy-classification model has not yet been established.

A substantial gap remains between detecting a physiological or behavioral change and classifying pregnancy reliably. Future studies should develop algorithms based on longitudinal wearable-sensor data and validate them at the animal level using independent cohorts from different farms, breeds, and physiological stages.

Small wearable devices require further improvements in comfort and durability to ensure long-term operation without causing discomfort to livestock. Battery life and data-transmission range also need to be optimized to meet the requirements of large-scale farms. With advances in the Internet of Things (IoT) and low-power wide-area network (LPWAN) technologies, devices based on LoRa technology and Arduino platforms have begun to be applied to livestock health monitoring [94,95]. These devices support remote data transmission and real-time monitoring and may provide more efficient solutions for large-scale farm management.

## 6. Conclusions

EPD has progressed from traditional empirical approaches to more precise diagnostic technologies. B-mode ultrasonography and appropriately validated PAG assays are established diagnostic methods, while P4 testing can support reproductive screening. In contrast, miRNAs, IRT, spectroscopy, machine vision, wearable sensing, and AI remain largely exploratory. Although these technologies may enable non-contact, automated, and high-throughput monitoring, current evidence does not support their use as stand-alone replacements for established methods. Multimodal AI may improve EPD by integrating complementary signals, but prospective, multicenter external validation using independent animals is required before routine implementation.

Future EPD systems are expected to become increasingly non-contact, low-stress, and high-throughput. Integrating wearable sensors, machine vision, time-series analysis, and AI may improve diagnostic robustness and operational efficiency. However, standardized data acquisition, appropriate reference standards, and validation under commercial farming conditions remain essential for reliable application.

## Figures and Tables

**Table 1:** Search sets used to identify studies on livestock EPD and related technologies.

Search Set	Purpose	Search String
**A**	Livestock EPD and diagnostic methods	(“pregnancy diagnos*” OR “pregnancy detect*” OR PAG OR P4 OR EPF OR Doppler OR IRT OR spectroscop* OR miRNA*) AND (livestock OR cattle OR bovine OR buffalo OR sheep OR ovine OR ewe OR goat OR caprine OR pig OR porcine OR sow OR rabbit OR heifer)
**B**	Machine vision and AI	(“machine vision” OR “computer vision” OR “deep learning” OR “artificial intelligence” OR “semantic segmentation”) AND (livestock OR cattle OR bovine OR buffalo OR sheep OR ovine OR ewe OR goat OR caprine OR pig OR porcine OR sow OR rabbit OR heifer) AND (pregnan* OR reproduction)
**C**	Wearable and multimodal sensing	(“wearable sensor*” OR “multimodal fusion” OR “sensor fusion” OR “time series” OR “optical sensing”) AND (livestock OR cattle OR bovine OR buffalo OR sheep OR ovine OR ewe OR goat OR caprine OR pig OR porcine OR sow OR rabbit OR heifer) AND (pregnan* OR reproduction)

Note. The search strings were adapted to the syntax of each database. Searches were conducted in the Topic field in Web of Science (TS), the title, abstract, and keyword fields in Scopus (TITLE-ABS-KEY), the title, abstract, or author-specified keyword fields in ScienceDirect, all metadata in IEEE Xplore, and the default search fields in Google Scholar. The asterisk indicates truncation where supported; explicit term variants were used in Google Scholar.

**Table 2:** Summary of representative methods for EPD in livestock.

Category	Method and Specimen/Examination	Representative Species	Timing After Breeding	Pregnancy Reference Standard	Validation Design	Reported Performance	Main Limitation	Reference
Physical/ behavioural	Transrectal palpation; manual rectal examination of the uterus	Egyptian buffalo	31–55 d; reliable from approximately d 46	PAG-RIA, using a pregnancy cutoff of at least 1.8 ng/mL	Paired within-study diagnostic evaluation	Sensitivity increased from 37.5% at d 31–35 to 93.8% at d 46–50 and 100% at d 51–55; specificity ranged from 90.9% to 100% (*n* = 168).	Late and operator-dependent; early examinations produce false negatives	[28]
Physical/ behavioural	Abdominal palpation; manual abdominal examination	New Zealand White rabbit	10.9 ± 0.3 d after mating	Subsequent kindling among does that carried to term	Single-study outcome-confirmed evaluation	All 20 does that subsequently carried to term were correctly identified; sensitivity and specificity for the complete cohort were not reported.	Small validation sample; operator-dependent and excessive pressure may cause stress or injury	[31]
Physical/ behavioural	Non-return to heat; behavioral observation	Red Sokoto goat	d 21	No independent reference standard; five pregnancy-assessment methods were compared	Comparative single-study evaluation without reference-standard diagnostic validation	Pregnancy proportions were 63.0%, 42.6%, and 73.6% across the three study groups; sensitivity, specificity, and overall accuracy were not estimated.	Indirect measure affected by oestrus expression and management; no independent accuracy estimate	[29]
Laboratory biomarker	P4 RIA; plasma progesterone measurement	Merino ewe	d 18 after AI	Subsequent lambing outcome	Single-study outcome-confirmed diagnostic evaluation	Sensitivity 100%, specificity 95.4%, PPV 81.6%, and NPV 100% at a cutoff of at least 1 ng/mL (*n* = 182).	Not pregnancy-specific; persistent luteal activity can cause false positives	[34]
Laboratory biomarker	EPF RIT; serum rosette inhibition test	Dairy cow	1–3 and 5–7 d after insemination	Rectal palpation at 42–45 d after insemination	Single-study diagnostic evaluation without a validated classification threshold	Significant discrimination was reported; sensitivity and specificity were not reported (23 inseminated cows and 18 controls).	Technically demanding assay, small study, and no validated diagnostic threshold	[39]
Laboratory biomarker	PAG latex strip; blood-based latex immunochromatography	Holstein cow	≥28 d after breeding	Transrectal B-mode ultrasonography at d 45 (*n* = 263)	Paired clinical evaluation within the same study; no external validation	Against ultrasonography in 263 cows: sensitivity 99.25%, specificity 90.00%, PPV 91.03%, NPV 99.15%, and accuracy 94.68%; 559 clinical samples were examined overall.	Residual PAG after pregnancy loss may cause false positives; concentration varies with parity and milk yield	[50]
Laboratory biomarker	Circulating miRNA; maternal-blood sequencing and qPCR	Buffalo	d18	Ultrasonography at d 35, followed by monitoring for 3–10 months	Exploratory within-study biomarker analysis; qPCR confirmation was not external diagnostic validation	Individual-miRNA AUCs ranged from 0.60 to 0.80 (30 pregnant and 20 nonpregnant buffaloes).	Requires a molecular laboratory; candidate panels and external validation are not standardized	[59]
Laboratory biomarker	Duplex ISG RT-qPCR (ISG15 and LGALS3BP); PBMC RNA	Buffalo	20 ± 2 d after AI	Color Doppler ultrasonography at d 40 after AI	Evaluation in two additional datasets within the same study; no multicenter external validation	Validation AUCs were 0.95 and 0.90; NPV ranged from 90% to 95% and PPV from 75% to 85%.	Requires RNA processing and qPCR; multicenter validation across breeds and management systems is needed	[63]
Imaging	B-mode ultrasonography; transabdominal scan using a 5-MHz probe	New Zealand White rabbit	Uterine fluid: 6.2 ± 0.2 d; fetal heartbeat: 7.8 ± 0.1 d	Subsequent kindling among does that carried to term	Single-study outcome-confirmed evaluation	All 20 does that subsequently carried to term were correctly identified; sensitivity and specificity for the complete cohort were not reported.	Small validation sample; image quality depends on operator, equipment, coupling, and preparation	[31]
Imaging	Luteal color Doppler and deep learning; transrectal Doppler videos	*Bos taurus* beef cow	d 20 and d 22 after FTAI	B-mode ultrasonography at d 29; embryo and uterine fluid were required	Animal-level restricted five-fold cross-validation (internal validation)	Deep-learning accuracy was 84–85% at d 20 and 86–89% at d 22; the best d 22 model achieved 99% sensitivity, 79% specificity, and 91% overall accuracy (*n* = 390).	Specialized ultrasound and model workflow; broader external validation remains necessary	[78]
Optical/thermal	IRT; eye, muzzle, flank, and vulval surface temperatures	Nili-Ravi buffalo	d 0–24 after AI, every 4 d	Transrectal B-mode ultrasonography at d 30	Retrospective within-study exploratory evaluation without a classification threshold	Site-specific temperature differences reached *p* ≤ 0.05; no diagnostic cutoff, sensitivity, or specificity was reported (*n* = 27).	Environmental and anatomical-site effects; a validated classification threshold is lacking	[3]
Optical/thermal	Vis-NIR spatial spectroscopy and SVM; abdominal diffuse-reflectance spectra	Rabbit	d 14 after AI	Not clearly reported in the cited study	Internal validation within the same study population	Validation set: sensitivity 93.18%, specificity 94.44%, and accuracy 93.88%; prediction set: 86.96%, 90.00%, and 90.69%, respectively.	Hair, skin thickness, probe placement, and dataset shift can affect spectra	[80]
Optical/thermal	RAPID multispectral sensing and SVM; eight-module abdominal optical array	Rabbit	d14 after AI	Not clearly reported in the cited study	Internal model evaluation plus cross-batch evaluation; no external validation	Primary analysis: accuracy 86.63% and recall 80.49% (*n* = 216); cross-batch accuracy declined to 78.36%.	Generalization varies with age and abdominal hair density	[26]

Performance values are reproduced from the cited studies and should not be compared directly across species, sampling windows, reference standards, or datasets. Abbreviations: AI, artificial insemination; AUC, area under the receiver operating characteristic curve; EPF, early pregnancy factor; FTAI, fixed-time artificial insemination; NPV, negative predictive value; PAG, pregnancy-associated glycoprotein; PBMC, peripheral blood mononuclear cell; PPV, positive predictive value; qPCR, quantitative polymerase chain reaction; RIA, radioimmunoassay; RIT, rosette inhibition test; SVM, support vector machine; Vis–NIR, visible–near-infrared.

## Data Availability

No new data were created or analyzed in this study.
